# Gentle Mind—A Podcast on Self-Compassion and Character Strengths to Enhance Well-Being in University Students: A Controlled Feasibility Study

**DOI:** 10.3390/bs16081332

**Published:** 2026-08-03

**Authors:** Marta Degani, Anna Flavia Di Natale, Giulia Cremaschi, Daniela Villani

**Affiliations:** 1 Department of Psychology, Università Cattolica del Sacro Cuore, 20123 Milan, Italy; giulia.cremaschi@unicatt.it; 2 Research Centre in Communication Psychology, Università Cattolica del Sacro Cuore, 20123 Milan, Italy; annaflavia.dinatale@unicatt.it (A.F.D.N.); daniela.villani@unicatt.it (D.V.)

**Keywords:** digital interventions, psychological well-being, self-compassion, character strengths, podcast, university students

## Abstract

Digital psychological interventions have emerged as accessible and scalable approaches for promoting mental well-being among university students. The present study evaluated the feasibility and preliminary efficacy of two interventions focused on self-compassion and character strengths among university students, a podcast-only intervention and a blended intervention combining podcasts with online group sessions, compared with a passive control group. Fifty-eight Italian university students were randomly assigned to a podcast intervention, a blended intervention, or a passive control group. Participants completed measures of self-compassion, perceived stress, and perfectionism at baseline, post-intervention, and one-month follow-up. Linear mixed models were used to examine changes over time and differences between groups. Results showed significant improvements in self-compassion in both intervention groups, with larger and more sustained effects in the blended condition. The blended intervention also produced significant reductions in perceived stress and maladaptive perfectionism standards, whereas the podcast-only intervention was associated with improvements primarily in self-compassion and delayed reductions in stress. No significant changes emerged in the control group. Descriptive data also revealed high levels of participants’ engagement and good acceptability of the intervention in both intervention groups, with marginally higher scores in the blended group. These findings suggest that podcast-based interventions may offer a feasible and accessible entry point to mental health support, while guided group activities may build on these initial gains to enhance well-being and reduce psychological distress.

## 1. Introduction

### 1.1. University Students’ Mental Health and Digital Psychological Interventions

The number of university students struggling with mental health concerns has doubled in the last decade and students are seeking help in greater numbers, with a growing severity of concerns (Lipson et al., 2022; Wang et al., 2021) and a heightened risk for the onset of mental health disorders and increased psychological distress related to academic, social, and financial pressures (Ferrari et al., 2022). Studies have shown that a substantial proportion of university students experience symptoms of depression, anxiety, and stress, while many face difficulties accessing traditional counselling services due to stigma, lack of time, or insufficient campus resources (Dale et al., 2026; Jacob & Maguvhe, 2026). The increasing need for accessible mental health support has prompted interest in scalable approaches capable of complementing traditional psychological services (Osborn et al., 2022).

In this context, digital psychological interventions have emerged as a promising strategy for promoting mental well-being and expanding access to evidence-based care. Digital psychological interventions refer to mental health and well-being support programs encompassing a broad range of formats that differ in both the technologies employed and the degree of human involvement (Pineda et al., 2023). They may include fully self-help interventions, such as mobile applications, web-based programs, online self-help materials or, more recently, artificial intelligence-based conversational agents; guided interventions, in which participants receive psychological support from a clinician, coach, or other facilitator; and blended interventions that combine digital components with structured human support. The digital content may be delivered asynchronously through formats such as web modules, prerecorded videos, or podcasts, or synchronously through messaging or videoconferencing (De Witte et al., 2021).

Digital psychological interventions have consistently demonstrated beneficial effects on psychological well-being and mental health outcomes across diverse populations (e.g., Cameron et al., 2025; Groot et al., 2023; Fu et al., 2020; Lehtimaki et al., 2021), including university students (Ferrari et al., 2022; Harith et al., 2022; Schäfer et al., 2024). Although important challenges remain, including suboptimal engagement (Baumel et al., 2019), relatively high attrition rates ranging from approximately 20% to 50% (Lewis et al., 2020; Richards & Richardson, 2012; Torous et al., 2020), and evidence largely derived from self-selected online samples that may limit generalizability (Baumel et al., 2019), digital interventions offer an accessible and scalable complement to campus mental health services. In particular, they have considerable potential to support prevention, facilitate early intervention, and expand access to evidence-based psychological care for students (Madrid-Cagigal et al., 2025; Lattie et al., 2019; Topooco et al., 2022).

Digital psychological interventions incorporate a variety of evidence-based approaches, including psychoeducation, mindfulness-based practices, cognitive-behavioral techniques, and stress-management strategies, with the aim of reducing psychological distress while promoting psychological well-being (Harith et al., 2022; Lattie et al., 2019). More recently, these interventions have increasingly integrated principles of positive psychology (Riva et al., 2012). Rather than focusing exclusively on symptom reduction, positive psychology-based interventions seek to promote flourishing by cultivating psychological resources and strengths that enable individuals to cope effectively with challenges and thrive (Biber & Davis, 2024; Saboor et al., 2024; Yurayat & Seechaliao, 2021). Which resources and strengths are most worth targeting, however, depends on the difficulties characteristic of the population served.

### 1.2. Self-Compassion and Character Strengths Digital Intervention Among University Students

Applied to the university context, this perspective suggests that interventions should target psychological resources that directly address the developmental and contextual challenges faced by students. Academic pressure, major life transitions, uncertainty about the future, and the need to balance multiple personal and academic responsibilities create an environment in which students are especially vulnerable to self-criticism, perfectionism, and diminished well-being (Madigan, 2019; McIntyre et al., 2018; Tunçel & Yalçın, 2026). Therefore, university students may particularly benefit from an approach grounded in positive psychology that emphasizes the identification and cultivation of personal strengths, as well as the development of a kinder and more supportive relationship with oneself. Among these approaches, interventions targeting self-compassion, as well as character strengths awareness and use, may be especially valuable for addressing the psychological challenges commonly experienced during university life.

Self-compassion, defined as treating oneself with kindness during times of difficulty, recognizing that suffering and imperfection are part of the shared human experience, and maintaining a balanced awareness of painful thoughts and emotions (K. Neff, 2003), has emerged as an important protective psychological resource. Among university students, higher levels of self-compassion have consistently been associated with greater psychological well-being, resilience, flourishing, and life satisfaction, as well as lower levels of depression, anxiety, stress, perfectionism, and self-criticism (e.g., Bluth et al., 2023; Koydemir & Sun-Selışık, 2016; Torres Lancheros et al., 2023).

On the other side, character strengths are relatively stable, positive personality traits reflected in an individual’s cognition, affect, and behavior, and are theorized within positive psychology as core components of a fulfilling life (Peterson & Seligman, 2004; Niemiec, 2014). Prior research has established a consistent association between character strengths and well-being among university students, linking them to higher life satisfaction, flourishing, and subjective and psychological well-being, alongside lower levels of stress, emotional distress, and depressive symptoms (Koydemir & Sun-Selışık, 2016; Cobo-Rendón et al., 2023; García-Álvarez et al., 2026).

Digital psychological interventions targeting self-compassion and character strengths have increasingly been developed to promote mental well-being, with both approaches showing preliminary encouraging results for university students; together, they can develop a practical strategy for managing and overcoming the challenges of their academic journey.

Several studies have examined the effectiveness of online self-compassion interventions in this population. Torres Lancheros et al. (2023) conducted an online Mindful Self-Compassion (MSC) intervention with 35 university students delivered synchronously in small groups through Google Meet and included psychoeducational materials and exercises. The findings indicated an increase in self-compassion and self-efficacy levels, alongside significant reductions in depression, stress, self-criticism, rumination, and worry. Consistent with these findings, Bluth et al. (2023) evaluated the feasibility and preliminary outcomes of a six-session online MSC program on a sample of 39 undergraduates or graduate students. The intervention was delivered live in an online group format over six weekly 1.5-h sessions. Results demonstrated enhanced self-compassion and emotional regulation skills, coupled with a decrease in stress and loneliness. Furthermore, González-García et al. (2021) investigated the feasibility of a mindfulness and self-compassion intervention among 66 university students during the COVID-19 pandemic. The intervention was delivered through an online platform combining psychoeducational mini-lectures, guided meditations, and reflective exercises. Results showed that the intervention was highly feasible and well accepted by students, with strong adherence and retention rates. Participants reported that the program helped them improve emotional regulation, self-awareness, concentration, and self-care during difficult moments. Importantly, significant reductions were observed in perceived stress and anxiety, while self-compassion levels increased significantly after the intervention.

Evidence supporting online character strengths interventions is also growing, although research specifically involving fully web-based programs for university students remains relatively limited. Significant insights were provided by Koydemir and Sun-Selışık (2016), who tested an 8-week online strengths-based intervention on 92 students in the seminar program. Results showed significant improvements in life satisfaction, subjective happiness, psychological health, and emotional well-being. Recent research by Zhu et al. (2024) compared Mindfulness-Based Strengths Practice (MBSP) with traditional Character Strengths-Based Interventions (CSBI). Both approaches were found to be effective, enhancing students’ well-being and their ability to apply personal strengths compared to a control group. Specifically, MBSP proved more effective in fostering mindfulness and psychological well-being, whereas CSBI was superior in enhancing subjective well-being. Additionally, Wong et al. (2024) conducted a single-session online CSBI focused on psychoeducation and the identification of personal strengths. While no significant inter-group differences were found, a significant time effect was observed for the experimental group regarding identity formation and reduction in self-doubt. Finally, a qualitative study by Kausar and Murryam (2025) on a two-week Zoom-based mindfulness and character strengths training revealed that students experienced a profound reconnection with themselves. Participants reported uncovering latent resources and talents previously obscured by academic routine. By visualizing their “best possible self,” students experienced intense emotional shifts, allowing them to perceive their intrinsic value beyond academic achievement. The integration of mindfulness with strengths analysis facilitated greater emotional awareness and led to concrete changes in daily habits.

However, to date, research has primarily examined digital self-compassion and character strengths interventions as distinct approaches. Yet these constructs may operate synergistically: self-compassion may reduce maladaptive self-evaluation in the face of academic challenges, while character strengths encourage students to mobilize their personal resources to thrive despite these challenges. Investigating their integration within a single digital intervention may therefore provide a more comprehensive approach to promoting mental well-being among university students.

### 1.3. Present Study

To address this gap, the present study investigated the feasibility and preliminary effectiveness of a digital intervention integrating self-compassion and character strengths to promote university students’ mental health and well-being.

To deliver this intervention, we selected the podcast format because its accessibility, flexibility, scalability, and growing popularity among young adults make it well suited to reaching university students, who often face time constraints, stigma, and other barriers to accessing conventional psychological support (Harith et al., 2022), enabling intervention content to be accessed privately and asynchronously during everyday activities such as commuting, exercising, or studying (Abas et al., 2020; Dascombe et al., 2025; Zou et al., 2025). Qualitative evidence supports this rationale, with students describing podcasts as accessible, anonymous, and supportive channels for mental health communication, valuing them for reassurance, psychoeducation, peer support, and emotional connection, and reporting that such content helps them feel understood and less isolated in their psychological distress (Riboldi et al., 2024).

Mental health podcasts typically deliver psychoeducation, mindfulness exercises, therapeutic discussions, lived-experience narratives, and evidence-based coping strategies in an on-demand audio format. Emerging evidence suggests that they can improve mental health knowledge, reduce stigma, increase willingness to seek professional help, and promote psychological well-being, with podcasts incorporating psychological exercises also showing promising effects on anxiety, perceived stress, and body image concerns (Caoilte et al., 2023; Carrotte et al., 2023; Denny et al., 2024). These benefits may partly stem from the conversational nature of podcasts, which combines expert knowledge with relatable narratives and practical advice, fostering emotional connection, perceived social support, and engagement with mental health topics (Carrotte et al., 2023; W. Li, 2026; Robins et al., 2024; Serini et al., 2025; Zou et al., 2025).

Research specifically examining podcast-delivered psychological interventions among university students remains limited but encouraging, showing promising effects on well-being and life satisfaction (Dreer, 2021; Karing, 2022). However, it remains unclear whether podcast listening alone is sufficient to promote meaningful psychological change (Turner-McGrievy et al., 2013; Zou et al., 2025). Growing evidence suggests that interventions incorporating human guidance or blended delivery formats may produce stronger and more sustained effects than entirely self-guided approaches, particularly for individuals experiencing greater psychological distress (Baños et al., 2022; Leung et al., 2022; Werntz et al., 2023), supporting the conceptualization proposed by Mohr et al. (2017), whereby digital mental health interventions are viewed as technology-enabled services rather than stand-alone products. For example, Karing (2022) compared a seven-week podcast-delivered mindfulness intervention with an equivalent facilitator-guided videoconference program. Both conditions produced significant improvements in mindfulness and life satisfaction, although reductions in depressive symptoms were greater in the guided condition. These findings suggest that podcast-based interventions may effectively promote well-being, while facilitator guidance and social interaction may provide additional benefits for students experiencing more pronounced psychological distress.

In light of these findings, the objective of the study was to test the preliminary efficacy of a podcast-based intervention tailored to university students (Dandil & Kingston, 2026) and designed to foster self-compassion and character strengths. Specifically, we compared a podcast-only format with a blended format combining the podcast with guided group videoconferences to examine their differential effects on students’ well-being relative to a passive control group.

Based on the study objectives, the following hypotheses were formulated:

**H1.**  *Both intervention groups (Podcast Intervention (PI) and Blended Intervention (BI)) would show significant improvements from baseline to post-intervention in self-compassion, perceived stress, and maladaptive perfectionism, and that these improvements would be maintained at follow-up.*

**H2.**  *The two intervention groups (PI and BI) and the passive control group would differ significantly in their longitudinal trajectories of self-compassion, perceived stress, and maladaptive perfectionism, with intervention participants showing greater improvements over time compared to controls.*

**H3.**  *Each intervention, the PI and BI, would produce significantly greater improvements in self-compassion, perceived stress, and maladaptive perfectionism compared to the passive control group, as reflected in their respective longitudinal trajectories.*

In addition to evaluating the intervention’s preliminary efficacy, the study also aimed to explore participants’ engagement with the intervention and to assess its acceptability. These outcomes were included to provide a broader understanding of the intervention’s feasibility, informing the future development and refinement of podcast-based positive psychology interventions for university students.

## 2. Materials and Methods

### 2.1. Study Design

The study had a mixed design with a comparison between three groups (two experimental groups and a passive control group) and three assessment moments: the baseline assessment (T0), the post-intervention assessment (T1) and the 4-week follow-up assessment (T2).

**Figure 1:**
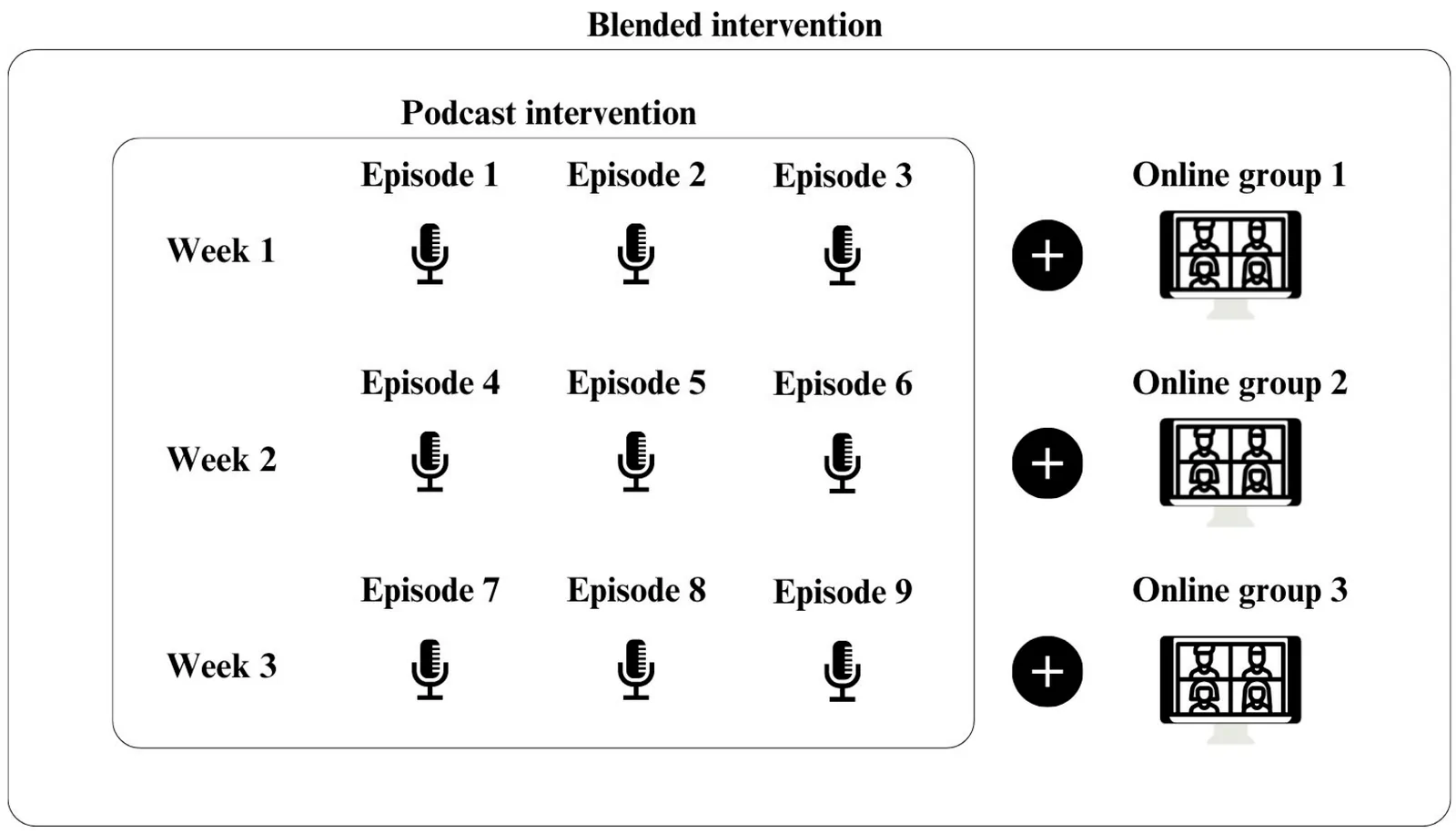
Structure of the podcast-based intervention (PI) and of the blended intervention (BI).

One experimental group underwent a PI while the other one participated in the same podcast-based intervention combined with three online group meetings (BI) as shown in Figure 1. This study was conducted from April 2024 to October 2025.

### 2.2. Participants

Participants were recruited through posts on researchers’ social profiles, online university courses and students’ associations notice boards and through word of mouth. As an approximation of the present analysis using linear mixed-effects models, the sample size of the feasibility study was calculated with G Power, and it was found that 54 participants would be needed to achieve 95% power with an average effect size (f = 0.25) with a repeated measurement design within/between interaction (number of groups 3; number of measurements: 3; correlation between repeated measurements: 0.5) (Faul et al., 2007).

To account for the possibility that not all interested students would complete the baseline assessment and enroll in the study, as well as for potential attrition during the intervention, a larger number of participants were initially recruited. A total of 105 university students expressed interest in participating in the study. Of these, 58 Italian university students completed the baseline assessment (T0). Participants had a mean age of 23.79 years (SD = 4.32), and 79% were female. They were recruited from different regions of Italy (47 from Northern Italy and 11 from Central and Southern Italy) and were enrolled in a range of degree programs (Education and Pedagogy = 13; Law = 11; Health Sciences = 11; Engineering and Hard Sciences = 10; Economics and Management = 7; Humanities = 6). Additionally, 23 participants were working students.

These participants were randomly assigned to one of the three study conditions using block randomization through a free online software (Research Randomizer 4.0). Nineteen were assigned to the PI, 18 were assigned to the BI and 21 were assigned to the control group. Retention rates differed across conditions. In the PI group, 19 participants completed the post-intervention assessment (T1) and 18 completed the follow-up assessment (T2). In the BI group, 14 participants completed T1 and 11 completed T2. In the control group, 21 participants completed T1 and 15 completed T2.

This study was approved by the Ethical Committee for Research in Psychology of the Department of Psychology of Università Cattolica del Sacro Cuore, protocol number 38/24.

### 2.3. Podcast Development

Podcast episodes focused on university students’ experience and distress through two positive psychology constructs, self-compassion (K. Neff, 2003; K. D. Neff, 2016) and character strengths (Peterson & Seligman, 2004). Self-compassion refers to the ability to be self-caring and compassionate in the face of difficulties and errors (self-kindness), to have a no-judgmental attention to one’s own feelings (mindfulness) and to understand that suffering is a part of everyone’s life (common humanity). This attitude has been used during the episodes to embrace failure experiences linked to university life and at the same time to promote a more functional approach to supporting motivation and career goals (Yang et al., 2023; Gunnell et al., 2017; Poots & Cassidy, 2020).

The character strengths model, on the other hand, was introduced to offer new ways of interpreting one’s own characteristics and uniqueness, with the aim of encouraging students to recognize and build upon their positive personal qualities, which they can use to overcome the challenges of the academic experience (Bellardita & Villani, 2024; García-Álvarez et al., 2023; Yu et al., 2022).

For the purposes of this study, a podcast called *Gentle Mind* (Degani, 2024) was specifically developed and published on Spotify to make it easily accessible to participants. The intervention consists of nine episodes of moderate length compared with those reported in the literature (Carrotte et al., 2025), each lasting between 9 and 14 min. This duration was chosen to balance the promotion of awareness and reflection with the feasibility of integrating the intervention into participants’ daily routines, in line with preferences reported among university students (Tam, 2012). Every week for three weeks, participants received three episodes to listen to whenever they wanted during the week.

Each episode is composed of a personal account describing a specific struggle experienced by a student (e.g., “I feel like a failure because I didn’t study enough today”) or the perspective of an expert professional (for two episodes). Building on the students’ contributions, the episode then focuses on exploring a specific aspect of the two psychological constructs on which the podcast is based, while also suggesting exercises or practical activities. Table 1 describes the contents of the podcast.

### 2.4. Podcast Intervention (PI) and Blended Intervention (BI)

The participants were divided into three groups: a passive control group and two experimental groups, one of which received the PI and the other received the BI. The PI required participants to listen to the 9 podcast episodes over the course of three weeks on their own. At the beginning and at the end of the three-week period, there were two brief group video calls to provide practical guidance and answer any questions that arose after the program concluded, without involving any program-related activities.

The BI also included participation in one weekly online group session lasting 1.5 h per week, for a total of 4.5 h of practical activities focused on the topics covered in each of the three episodes of the week, in order to not be too time engaging as another study on the same population with 1 online group session per week about 2 h each ones (Torres Lancheros et al., 2023). The intention of this second experimental group was to propose a blended intervention composed by a self-help and psychoeducational based (the podcast) with three online meetings with other students with similar issues and an expert with psychological education to apply the knowledge and insights gained from listening to the podcast and to integrate and to internalize new strategies for dealing with academic challenges; as suggest also by Zou et al. (2025) to try to improve the change in addition to awareness.

The first meeting focused on self-criticism and self-compassion to propose practical applications and examples of the content in episodes 1, 2 and 3 through the following activities: *Write a compassionate letter to a self-critical student who is facing difficulties at university. Transform self-critical and perfectionist sentences into supportive and Self-Compassion versions. Visualize a compassionate figure in a safe place with group sharing*. The second session focused on cognitive defusion, drawing on the content of episodes 4, 5, and 6 through these activities: *brainstorming about locus of control “what I can control and what I can’t in University” and guided breathwork and group sharing*. The last group meeting focused on self-enhancement based on the 7, 8 and 9 episodes through these activities: *Share and comment on your five signature strengths. Choose an action to improve one Character Strength. Use one of your signature strengths to cope with a difficult.*

### 2.5. Measures

The two groups completed the following online questionnaires on Qualtrics at T0, T1 and T2 assessment moments.

#### 2.5.1. Socio-Demographic Information

Information was requested regarding gender, age, profession, nationality, educational qualification, and region of residence. This measure was administered at T0.

#### 2.5.2. Mental Well-Being

##### Self-Compassion (SC)

In the present study we used the Self-Compassion Scale designed by K. D. Neff (2003; Italian version: Veneziani et al., 2017). It is composed of 24 items divided into three positive sub-scales: Self-kindness, Mindfulness and Common Humanity; and three negative sub-scales: Self-judgment, Over-identification and Isolation. Each item can be answered through a 5-step Likert scale where 1 means “almost never” and 5 means “almost always”. The reliability of the scale for this study was good (α = 0.90 and ω = 0.90 for this study). This measure was administered at T0, T1 and T2.

#### 2.5.3. Mental Distress

##### Perceived Stress (PS)

In the present study we used the Italian Perceived Stress Scale-4, developed by Cohen and Williamson (1988, Italian version: Mondo et al. 2021), which aims to measure the perceived stress in the last two weeks. It is composed of 4 items (two straight and two reverse) on a 5-step Likert scale where 0 means “never” and 4 means “always”. Total score is calculated by adding up the single items score, the range is from 0 to 16. The reliability of the scale for this study was acceptable (α = 0.68 and ω = 0.70). This measure was administered at T0, T1 and T2.

##### Perfectionism (PF)

In the present study we used the Short Almost Perfect Scale, developed by Rice et al. (2014; Italian version: Loscalzo et al., 2019) which measures perfectionism levels in two separated sub-scale: one that explores the presence of expectations for high-quality performance (Standards, PF-S) (α = 0.82 and ω = 0.83 for this study) and one that explores self-critical attitudes when there is a perceived gap between ideal performance and actual performance achieved (Discrepancy, PF-D) (α = 0.87 and ω = 0.87 for this study). The scale is composed of 8 items on a 7-step Likert scale where 1 means “totally agree” and 7 “totally disagree”. This measure was administered at T0, T1 and T2.

#### 2.5.4. Participants’ Engagement and Acceptance of the Intervention

##### Participants’ Engagement

Participants’ engagement with the podcast intervention was assessed at T1 by asking participants to indicate which podcast episodes, if any, they had not listened to and to provide the reasons using an open-ended response. Attendance at the guided group sessions in the blended intervention was monitored using attendance registers.

##### Intervention Acceptance

At T1, participants in both experimental groups completed a questionnaire assessing the acceptability of the intervention. Specifically, participants rated the pleasantness of the podcast (“How pleasant was listening to the podcast?”) and their overall satisfaction with the podcast (“How likely would you be to recommend the podcast to a friend?”). Participants also evaluated the usefulness and ease of the activities included in the intervention. For participants in the PI group, these items referred to the activities proposed within the podcast episodes. For participants in the BI group, they referred to the activities introduced through the podcast and subsequently completed during the group sessions. Finally, participants evaluated their perceived self-awareness increase (“To what extent do you think the podcast content helped you better understand yourself and how you function in everyday situations?”). All items were evaluated using a 7-point Likert scale ranging from 1 (not at all) to 7 (very much).

### 2.6. Data Analyses

First, preliminary analyses were conducted to examine potential baseline differences between groups across all outcome variables. Separate one-way analyses of variance (ANOVAs) were performed to assess group differences at baseline in self-compassion (SC), perceived stress (PS), perfectionism standards (PF-S), and perfectionism discrepancy (PF-D).

To examine intervention effects, separate Linear Mixed Models (LMMs) were conducted for each outcome variable (self-compassion, perceived stress, perfectionism standards, and perfectionism discrepancy). In all models, group (PI, BI, control), time (T1, T2, T3), and their interaction were included as fixed effects, while participant-specific random intercepts were specified to account for the dependency of repeated observations nested within individuals. LMMs were selected because they are particularly appropriate for longitudinal repeated measures designs with unequal group sizes and missing data across time points, as they allow the inclusion of all available observations without excluding participants with incomplete assessments, thereby reducing bias associated with listwise deletion.

Following the LMMs, two sets of planned contrasts were conducted. The first set directly compared the longitudinal trajectories of PI and BI with the passive control group, testing whether changes from baseline to post-intervention (T0–T1) and from baseline to follow-up (T0–T2) were significantly greater in each active condition than in the control. The second set examined within-group changes over time, testing changes from T0 to T1 and from T0 to T2 separately within the PI, BI, and control groups, in order to evaluate immediate intervention effects and the maintenance of gains over time.

Taken together, these analyses addressed the three study hypotheses: the LMM interaction tested whether the groups followed distinct trajectories (H2); the between-group contrasts tested whether each intervention outperformed the passive control (H3); and the within-group contrasts tested whether improvements emerged and were sustained within each condition (H1).

In addition, participants’ engagement with and acceptance of the intervention were examined descriptively. Engagement was summarized using the frequency of podcast listening in both intervention groups and attendance at the guided group sessions in the BI group. Intervention acceptance was described using the means and standard deviations of the ad hoc items assessing podcast pleasantness, overall satisfaction, perceived usefulness and ease of the intervention activities, and perceived increase in self-awareness.

## 3. Results

### 3.1. Baseline Differences Between Groups Across All Outcome Variables

Means and standard deviations in SC, PS, PF-S, and PF-D for all groups and all time periods are given in Table 2. To control for differences in outcome variables at baseline, we conducted a one-way ANOVA with the three groups for each outcome variable: SC, PS, PF-S, and PF-D. The analysis revealed no statistical differences at baseline (all ps > 0.05).

### 3.2. Intervention Effect

Results are organized by construct, and for each construct, findings are presented with reference to the three hypotheses formulated in the study. Detailed results of the planned between-group and within-group contrasts, including regression estimates, standard errors, 95% confidence intervals, and *p*-values, are provided in Appendix A.

#### 3.2.1. Self-Compassion

The LMM for SC revealed a significant main effect of time, F(2, 93.26) = 20.60, *p* < 0.001, and a significant Group × Time interaction, F(4, 93.11) = 3.22, *p* = 0.016, but no significant main effect of group, F(2, 54.75) = 1.79, *p* = 0.177. The model explained 79% (conditional R^2^ = 0.79).

Between-group contrasts indicated that the BI group showed significantly greater improvements in self-compassion than the control group both from T0 to T1 (β = 0.54, SE = 0.16, *p* < 0.001) and from T0 to T2 (β = 0.41, SE = 0.18, *p* < 0.05). In contrast, improvements in the PI group did not differ significantly from the control group at either time point (all ps > 0.05).

Within-group analyses showed significant increases in self-compassion over time in both intervention groups (PI: T0–T1, β = 0.31, *p* < 0.01; T0–T2, β = 0.46, *p* < 0.001; BI: T0–T1, β = 0.56, *p* < 0.001; T0–T2, β = 0.60, *p* < 0.001), whereas no significant changes were observed in the control group.

#### 3.2.2. Perceived Stress

The LMM for PS revealed a significant main effect of time, F(2, 94.29) = 7.55, *p* < 0.001, but no significant main effect of group, F(2, 55.43) = 1.86, *p* = 0.165, and no significant Group × Time interaction, F(4, 94.14) = 2.05, *p* = 0.094. The model explained 74% of the variance (conditional R^2^ = 0.74).

Between-group contrasts showed that the BI group reported significantly greater reductions in perceived stress than the control group from T0 to T1 (β = −1.81, SE = 0.72, *p* < 0.05), whereas the corresponding comparison at T2 approached significance (*p* = 0.060). The PI group did not differ significantly from the control group at either assessment.

Within-group analyses indicated significant reductions in perceived stress in the BI group at both T1 (β = −1.57, SE = 0.55, *p* < 0.01) and T2 (β = −1.81, SE = 0.61, *p* < 0.01), whereas the PI group showed a significant reduction only at follow-up (β = −1.54, SE = 0.49, *p* < 0.01). No significant changes emerged in the control group.

#### 3.2.3. Perfectionism-Standards

The LMM for PF-S revealed a marginally significant main effect of time, F(2, 95.37) = 3.08, *p* = 0.050, but no significant main effect of group, F(2, 56.18) = 0.51, *p* = 0.602, and no significant Group × Time interaction, F(4, 95.18) = 2.27, *p* = 0.067. The model explained 69% of the variance (conditional R^2^ = 0.69).

Between-group contrasts showed that only the BI group demonstrated significantly greater reductions in perfectionistic standards than the control group at post-intervention (β = −0.93, SE = 0.32, *p* = 0.004), whereas the T2 comparison approached significance (*p* = 0.069). No significant differences emerged for the PI group.

Within-group analyses confirmed significant reductions only in the BI group (T0–T1: β = −0.73, SE = 0.25, *p* < 0.01; T0–T2: β = −0.72, SE = 0.27, *p* < 0.01), while neither the PI nor the control group showed significant changes.

#### 3.2.4. Perfectionism-Discrepancy

The LMM for PF-D revealed no significant main effect of time, F(2, 95.08) = 2.37, *p* = 0.099, no significant main effect of group, F(2, 55.49) = 0.33, *p* = 0.722, and no significant Group × Time interaction, F(4, 94.86) = 0.86, *p* = 0.490. The model explained 64% of the variance including random effects (conditional R^2^ = 0.64).

Between-group contrasts indicated that neither intervention differed significantly from the control group.

Within-group analyses showed a marginal reduction in perfectionistic discrepancy in the BI group from T0 to T1 (β = −0.65, SE = 0.33, *p* = 0.054) and a significant reduction in perfectionistic discrepancy only in the BI group at follow-up (β = −0.82, SE = 0.36, *p* < 0.05), whereas all remaining comparisons were non-significant.

#### 3.2.5. Participants’ Engagement Outcomes

Participants’ engagement was assessed through adherence to podcast listening and participation in the online group session activities. In the PI group, only three participants listened to 8 of the 9 podcast episodes (89%), reporting lack of time as the reason for missing one episode. In the BI group, one participant listened to 8 of the 9 episodes (89%) and another listened to 7 of the 9 episodes (78%), both due to lack of time. The participation in group sessions was registered by the researcher with 100% attendance and participation.

#### 3.2.6. Intervention Acceptance Outcomes

Regarding intervention acceptance, participants in both intervention groups reported high levels of podcast pleasantness (PI: M = 5.89, SD = 0.57; BI: M = 6.31, SD = 1.11) and overall satisfaction (PI: M = 6.11, SD = 1.15; BI: M = 6.54, SD = 0.78), with consistently higher ratings in the BI group. Similarly, participants perceived the intervention activities as useful (PI: M = 5.79, SD = 1.16; BI: M = 6.54, SD = 0.71) and easy to complete (PI: M = 4.53, SD = 0.96; BI: M = 6.15, SD = 0.90), again with more favorable evaluations in the BI group. Finally, participants in both groups reported a high perceived increase in self-awareness following the intervention, with higher ratings in the BI group (PI: M = 5.63, SD = 1.07; BI: M = 6.23, SD = 1.17).

## 4. Discussion

The aim of this study was to evaluate the feasibility and preliminary efficacy of two digital psychological interventions designed to promote mental well-being among university students, specifically, a podcast-based intervention (PI) and a blended intervention (BI) combining podcast content with guided activities, by comparing them to a passive control group. Both protocols focused on self-compassion and character strengths, two positive psychology constructs widely recognized for fostering student well-being (Bluth et al., 2023; González-García et al., 2021; Koydemir & Sun-Selışık, 2016; Torres Lancheros et al., 2023). The first hypothesis (H1), addressing within-group changes, was largely supported. Both interventions produced significant and sustained improvements in self-compassion from baseline to post-intervention that were maintained at follow-up, whereas the control group showed no significant change. This finding is consistent with previous research demonstrating that online self-compassion interventions enhance self-compassion while reducing self-critical and distress-related outcomes (Bluth et al., 2023; González-García et al., 2021; Torres Lancheros et al., 2023). Improvements in perceived stress differed across conditions: the BI group showed significant reductions immediately after the intervention that were maintained at follow-up, whereas the PI group showed a delayed improvement that emerged only at follow-up. This pattern suggests that self-guided podcast interventions may require additional time for participants to integrate and apply the proposed strategies in everyday life, consistent with evidence that unguided digital interventions often produce slower effects than guided approaches (Amanvermez et al., 2022; Fischer et al., 2020). Finally, only the BI group showed improvements in perfectionism, with reductions in perfectionism standards evident at both post-intervention and follow-up, and a more gradual reduction in perfectionism discrepancy that became significant only at follow-up. These findings suggest that modifying perfectionistic cognitions may require greater behavioral engagement and guided practice than can be achieved through passive podcast listening alone. This is theoretically coherent with the multidimensional model of perfectionism (Slaney et al., 2001; Rice et al., 2014): the discrepancy dimension captures a more stable and deeply rooted self-critical evaluative process than the standards dimension, and therefore likely demands more intensive or prolonged intervention to produce meaningful change.

The second (H2) and third (H3) hypotheses examined between-group differences. Overall, the BI consistently outperformed the PI condition. For self-compassion, participants in the BI group showed significantly greater improvements than the control group at both post-intervention and follow-up, whereas the PI group did not differ significantly from the control group. A similar pattern emerged for perceived stress, although between-group differences were observed only immediately after the intervention. For perfectionism, significant between-group effects were limited to perfectionism standards, with the BI group showing greater reductions than the control group at post-intervention only. No significant between-group differences emerged for perfectionism discrepancy. Taken together, these findings suggest that while podcast-based interventions may promote beneficial within-person changes, integrating podcasts with guided group activities appears to enhance their effectiveness, particularly for outcomes requiring greater reflection, practice, and interpersonal support.

The absence of significant between-group effects for the podcast intervention should not be interpreted as evidence of ineffectiveness. As indicated by the within-group analyses, participants in the PI condition showed sustained improvements in self-compassion and a delayed reduction in perceived stress at follow-up, suggesting that podcast-based interventions may foster gradual psychological change that is less readily detectable in between-group comparisons. This interpretation is consistent with previous research indicating that podcasts are particularly effective for increasing psychological awareness, self-reflection, and mental health literacy, whereas broader emotional and behavioral changes may require more time or additional support to emerge (Dreer, 2021; Turner-McGrievy et al., 2013). The conversational nature of podcasts may facilitate emotional engagement and perceived social connection (Denny et al., 2024; W. Li, 2026; Serini et al., 2025), but meaningful psychological change likely depends on listeners actively integrating the proposed strategies into their daily lives (Carrotte et al., 2025; Zou et al., 2025). Accordingly, podcast-only interventions may be best conceptualized as accessible, low-intensity resources for promoting self-awareness and early psychological skill development rather than as stand-alone alternatives to more interactive interventions.

By contrast, the BI produced broader and more immediate improvements across outcomes. This finding is consistent with Karing (2022), who similarly reported greater reductions in distress following a guided videoconference mindfulness intervention than a podcast-only format, and with evidence showing that human support enhances the effectiveness of digital interventions (Cuijpers et al., 2019; Karyotaki et al., 2021; Mohr et al., 2011, 2017). Meta-analytic evidence suggests that guided interventions generally outperform self-guided approaches (Baumeister et al., 2014; Richards & Richardson, 2012), with their effectiveness depending not only on the presence of human guidance but also on its quality and implementation (Mira et al., 2017). According to the Supportive Accountability model (Mohr et al., 2011, 2017), guidance can increase engagement by providing accountability, encouragement, and opportunities to clarify and apply intervention content. From this perspective, blended interventions may represent a particularly promising approach, combining the flexibility and scalability of self-guided digital content with the benefits of structured, high-quality professional support (Rheker et al., 2015). In the present study, the group meetings likely served this function by enabling participants to discuss the podcast material, practice self-compassion and character strengths in relation to their own experiences and receive support from both peers and the facilitator. This interpretation is also reflected in the feasibility findings: although engagement and acceptance were high in both intervention groups, participants in the BI condition consistently reported greater pleasantness, usefulness, ease of the activities, satisfaction, and perceived gains in self-awareness. While these findings are descriptive, they suggest that combining podcasts with guided group activities may enhance participants’ experience and the intervention’s overall impact.

These considerations may be particularly relevant in the context of the rapid expansion of artificial intelligence–based conversational agents as digital mental health tools (H. Li et al., 2023). Although conversational AI may substantially increase scalability and accessibility, recent work has emphasized that AI-generated empathy should not be equated with genuine human empathy and that meaningful human interaction remains an essential component of psychological support (Lin et al., 2024).

Together, these findings highlight a central trade-off between accessibility and effectiveness. The podcast-only intervention offers a highly flexible, scalable, and low-barrier approach that may be particularly suitable for students reluctant or unable to engage in more intensive psychological support. Its asynchronous, flexible, and non-intrusive format likely contributed to the high retention observed in the present study by reducing organizational burden and facilitating spontaneous engagement (Andersson & Jaldemark, 2026). Moreover, standalone podcast interventions have been shown to raise mental health awareness, foster self-reflection, and provide meaningful psychological benefits while requiring minimal active commitment and preserving participant anonymity (Riboldi et al., 2024), characteristics that may be especially valuable for university students facing time constraints or concerns about stigma. In contrast, the blended intervention appears to maximize psychological benefits by integrating the accessibility of podcasts with opportunities for guided practice, reflection, and social interaction. At the same time, its greater organizational demands were accompanied by higher attrition, suggesting that increased effectiveness may come at the cost of reduced reach.

This pattern supports a stepped-care perspective, whereby podcast-only interventions may function as an accessible entry point to mental health support, encouraging students to recognize their psychological needs, reflect on their experiences, and become aware of available support options. Blended interventions may extend these initial gains through guided practice, interpersonal interaction, and increased behavioral activation, thereby promoting deeper learning and more enduring psychological change. Integrating both formats into university mental health services may therefore provide a scalable and flexible continuum of care, enabling students to access support that matches their needs while optimizing the reach and effectiveness of campus-based mental health provision. Such an approach helps address the well-documented gap between students’ mental health needs and their use of available services by providing multiple, graduated pathways to care that accommodate different levels of readiness and engagement (Osborn et al., 2022).

### Strengths, Limitations and Future Directions

The present study has several notable strengths. First, the intervention itself represents a methodological strength: the Gentle Mind podcast was purpose-built for the study, grounded in evidence-based theoretical models, and specifically tailored to the experience and challenges of university students, rather than relying on pre-existing commercial content. Second, the inclusion of two active experimental conditions, a podcast-only and a blended format, alongside a passive control group allowed for a more nuanced assessment of intervention effects than a simple treatment versus control design, enabling direct comparison of two delivery formats differing in the degree of human support and active engagement. Third, the three-timepoint design, with assessments at baseline, post-intervention, and one-month follow-up, allowed not only the evaluation of immediate effects but also the examination of whether gains were maintained over time, which is rarely addressed in feasibility studies of this kind.

However, this study has several limitations that should be considered when interpreting the findings. First, participants were recruited through online advertisements, university networks, and voluntary participation, resulting in a convenience sample. Consequently, self-selection bias may have occurred, as individuals with a greater interest in mental health, self-compassion, or positive psychology interventions may have been more likely to participate. This limitation is common in research (Hiratsuka, 2025) and may reduce the representativeness of the sample. In line with previous studies (Caldarelli et al., 2024), the sample was predominantly female (79%), which limits the generalizability of findings to the broader university student population and raises the question of whether the effects observed would replicate in more gender-balanced samples. This issue is particularly relevant because previous research has shown gender differences in university students’ psychological distress, perceived stress, coping strategies, and help-seeking behaviors. For example, female students often report higher levels of stress, anxiety, depressive symptoms, and emotional coping than male students, whereas male students may be less likely to disclose psychological difficulties or seek support due to gendered norms around emotional expression and help-seeking (Graves et al., 2021; Prowse et al., 2021; Porru et al., 2021). These differences may influence both baseline levels of distress and engagement with psychological or well-being interventions. Consequently, a sample with a high proportion of women may overrepresent students who are more willing to participate in, engage with, or benefit from such interventions. Future studies should therefore aim to recruit more gender-balanced and gender-diverse samples and should test gender as a potential moderator of intervention outcomes. This would clarify whether the observed benefits are robust across student subgroups or whether intervention effects vary as a function of gender.

A further limitation concerns the use of a passive control group. Although passive or waitlist control conditions are common in psychological intervention research, they provide a relatively weak comparison because they do not control for non-specific factors such as expectancy effects, attention, perceived credibility of the intervention, demand characteristics, or the mere act of repeated assessment. This issue is consistent with previous meta-analytic evidence showing that intervention effects are often larger when psychological interventions are compared with passive rather than active control groups (Dawson et al., 2020; Han & Kim, 2023; Wakelin et al., 2022). Future research would therefore benefit from including an active control condition matched to the intervention groups in duration, format, researcher contact, and perceived credibility. For example, participants in the control condition could listen to podcasts or audio materials on neutral academic topics, study skills, or general university information (e.g., Smeets et al., 2014) that do not directly target self-compassion, character strengths, or emotional well-being.

An additional limitation concerns the higher attrition observed in the blended intervention group. This issue is especially relevant in digital and blended psychological interventions, where adherence and retention are recognized challenges. Systematic and meta-analytic evidence shows that online and smartphone-delivered mental health interventions frequently experience substantial attrition and declining engagement over time (Alrashdi et al., 2024; Linardon & Fuller-Tyszkiewicz, 2020). The higher dropout in the blended condition may therefore reflect the additional demands associated with this format. Compared with a fully self-guided intervention, blended interventions often require not only individual completion of online materials but also attendance at live or group-based sessions. These added components may increase structure, social support, and accountability, but they can also create barriers related to scheduling, time burden, social discomfort, or competing academic commitments. Future studies should therefore examine the reasons for dropouts more directly, for example by collecting brief exit feedback from participants who discontinue the intervention. They should also consider design strategies aimed at reducing burden while preserving the benefits of blended delivery. These may include, for example, flexible scheduling, asynchronous alternatives to live group sessions, shorter or modular intervention components, reminder systems or brief check-in contacts. Such strategies are supported by evidence suggesting that reminders, compensation, and engagement support may reduce attrition in digital mental health interventions (Linardon & Fuller-Tyszkiewicz, 2020). This is particularly relevant considering that participant attrition reduced sample size at follow-up, limiting the interpretability of the results.

Finally, a limitation concerns the interpretation of the planned contrasts. Although the study hypotheses specified a priori comparisons between intervention conditions and the control group, several findings emerged in the absence of a significant overall Group × Time interaction. Planned contrasts are statistically justifiable when theoretically driven and specified in advance, and they may provide more focused tests of specific hypotheses than omnibus tests, particularly in factorial and mixed-model designs (Granziol et al., 2025; Schad et al., 2020). Moreover, omnibus and contrast-based tests do not always yield identical conclusions, and a non-significant omnibus test does not necessarily preclude meaningful focused comparisons (Chen et al., 2018). Nevertheless, such findings should be interpreted with caution. Significant contrasts in the context of non-significant omnibus interaction effects may reflect more localized or modest intervention effects and should be considered less robust than effects supported by both omnibus and follow-up tests. Furthermore, the relatively small sample size may have limited statistical power to detect interaction effects consistently across outcomes. Consequently, the observed pattern of results should be regarded as preliminary and in need of replication in larger, adequately powered trials before firm conclusions can be drawn regarding the effectiveness of the intervention.

## 5. Conclusions

The present study evaluated the feasibility and preliminary efficacy of two podcast-based psychological interventions designed to promote mental well-being among university students, comparing a podcast-only format and a blended format combining podcast content with guided group sessions against a passive control group. The findings indicate that both interventions produced meaningful improvements in self-compassion, while the blended intervention showed broader and more robust effects, extending to perceived stress and maladaptive perfectionism. These findings suggest that podcast-based interventions may represent a feasible and accessible way of delivering psychoeducational content and promoting self-compassion among university students. However, the broader and more robust effects observed in the blended intervention indicate that podcasts may be most effective when integrated with opportunities for guided human interaction and group discussion, rather than as a substitute for interpersonal support.

At the same time, the two formats appear to serve complementary rather than competing functions. The podcast-only intervention offers a flexible, anonymous, and low-commitment resource capable of reaching a broad audience with minimal organizational burden, making it particularly suitable as a first-line tool for awareness promotion and self-reflection. The blended intervention, while more demanding, may be better suited for students seeking deeper or more sustained psychological change.

Specifically, podcasts could therefore represent a psychoeducational tool to be made accessible to the student population, promoting self-reflection and the management of challenges typical of both this life cycle stage and the academic journey. Nevertheless, in a historical moment marked by deep youth vulnerability (Ferrari et al., 2022; Lipson et al., 2022; Wang et al., 2021) and a severe strain on counseling centers struggling to meet a skyrocketing demand for mental health services, colleges are still searching for novel solutions (Taylor et al., 2024). In this regard, a blended podcast intervention seamlessly inserts itself into these needs, integrating with the traditional path of the professional to support those in need. By combining independent spaces for self-reflection with professional guidance, this approach leverages and actively sustains the implementation and maintenance of cost-effective digital solutions developed to address this vulnerability.

Taken together, the present findings contribute to the growing evidence base for digital well-being interventions in higher education and highlight the potential of podcasts as an innovative and accessible medium for delivering evidence-based psychological content to university students.

## Figures and Tables

**Table 1:** Podcast contents.

Podcast Episode Titles	Podcast Contents
1. The reason we are self-critical	Explanation of the relationship between self-criticism and threat defense mechanism (Gilbert & Petrocchi, 2012) and presentation of Self-Compassion as functional alternative (C. Germer & Neff, 2021). *Suggested activities*: Reflection about own self-criticism’s motivation. Compassionate hug practice.
2. Our inner dialogue	Explanation of what the inner dialogue is, its impact on emotion and performance and how to change it in a more supportive and gentle voice. *Suggested activities*: Reflection about own inner dialogue’s characteristics. Track and change own critical inner dialogue (Viou & Georgaca, 2021).
3. Accept failure and everything that comes with it	Normalization of failure experience and acceptance of difficult emotions (Barrett et al., 2001) with awareness and Self-Compassion *Suggested activities*: Compassionate figure in the safe place visualization (Malighetti et al., 2023). Build your own compassionate mantra.
4. Personal value does not depend on results	Discussion of the misconception that personal value depends on perfection and academic success and the fear that being Self-Compassionate negatively impacts personal and academic realization. *Suggested activity*: Write a compassionate letter to yourself, underlying that your value is independent from your actions or your results (C. K. Germer & Neff, 2013).
5. Take responsibility without being self-critical	The episode deals with the concept of locus of control (Ajzen, 2002) emphasizes the difference between constructive responsibility and self-critical fault. *Suggested activity*: Self-Compassionate reflection about a real personal failure experience.
6. Awareness of the present moment	Explicit exploration of Mindfulness in the Self-Compassion approach as a strategy to manage the default mode network (Raichle, 2015) and the cognitive overload caused by self-critical and perfectionist rumination. *Suggested activity*: Guided breathwork and Mindful shower or walk.
7. Shifting from constraints to resources	Reflection about the importance of emphasizing own qualities and resources rather than weaknesses in approaching the job market. Proposing the Character Strengths model as a lens through which to observe oneself and one’s own educational and growth path. *Suggested activity*: Complete VIA questionnaire (Niemiec, 2012).
8. Character strengths in university	Explanation of how to apply and recognize personal Character Strengths and reflection about the risk of overuse according to the VIA model. *Suggested activity*: exercises to integrate and apply Character Strengths in daily life.
9. Follow your own path	Conclusion of the intervention focused on the importance of making decisions aligned with one’s personal desire and characteristics rather than based on external judgment or expectation. Recalling the Self-Compassion combined with Character Strengths as supportive tools in academic experience.

**Table 2:** Groups’ means and standard deviation in T0, T1 and T2.

		PI			BI			Control		
	Range (Score)	T0 (*n* = 19)	T1 (*n* = 19)	T2 (*n* = 18)	T0 (*n* = 18)	T1 (*n* = 14)	T2 (*n* = 11)	T0 (*n* = 21)	T1 (*n* = 21)	T2 (*n* = 15)
SC	1–5 (mean)	2.71 (0.37)	3.02 (0.55)	3.18 (0.50)	2.68 (0.61)	3.32 (0.64)	3.48 (0.61)	2.63 (0.81)	2.65 (0.86)	3.04 (0.79)
PS	0–4 (sum)	7.89 (1.76)	7.37 (2.77)	6.39 (2.28)	8.17 (3.01)	6.79 (2.01)	6.00 (2.53)	8.48 (3.57)	8.71 (3.18)	7.33 (2.77)
PF-S	1–7 (mean)	5.24 (1.15)	5.17 (1.27)	5.04 (1.14)	5.88 (1.16)	5.07 (1.16)	5.07 (0.91)	5.40 (1.33)	5.61 (1.13)	5.25 (1.09)
PF-D	1–7 (mean)	4.22 (1.28)	3.95 (1.64)	4.37 (1.12)	4.49 (1.74)	3.77 (1.74)	3.61 (1.50)	4.37 (1.51)	4.29 (1.53)	4.10 (1.64)

## Data Availability

The data that support the findings of this study are available from the corresponding author upon reasonable request.

## Abbreviations

ANOVAS	Separated one-way analyses of variance
BI	Blended Intervention
CSBI	Character Strengths-Based Interventions
H1, H2, H3	Hypothesis 1, 2 and 3
MBSP	Mindfulness-Based Strengths Practice
LMMs	Linear Mixed Models
PI	Podcast Intervention
PF	Perfectionism
PF-D	Perfectionism Discrepancy dimension of Short Almost Perfect Scale
PF-S	Perfectionism Standard dimension of Short Almost Perfect Scale
PS	Perceived Stress
SC	Self-compassion
T0	Baseline assessment
T1	Post-intervention assessment
T2	Follow-up assessment

## Appendix A. Full Results of Planned Between-Group and Within-Group Contrasts from the Linear Mixed Models

**Table A1:** Planned between-group contrasts from the linear mixed models. Estimates (β), standard errors (SE), 95% confidence intervals (CI), and *p*-values comparing changes in the intervention groups relative to the control group.

Outcome	Comparison	β	SE	95% CI	*p*
Self-compassion	PI vs. Control (T0–T1)	0.28	0.15	−0.00, 0.57	0.057
	PI vs. Control (T0–T2)	0.27	0.16	−0.04, 0.58	0.093
	BI vs. Control (T0–T1)	0.54	0.16	0.23, 0.85	<0.001
	BI vs. Control (T0–T2)	0.41	0.18	0.06, 0.75	<0.05
Perceived Stress	PI vs. Control (T0–T1)	–0.76	0.67	–2.07, 0.54	0.255
	PI vs. Control (T0–T2)	–1.25	0.72	–2.66, 0.16	0.085
	BI vs. Control (T0–T1)	–1.81	0.72	–3.22, –0.39	<0.05
	BI vs. Control (T0–T2)	–1.52	0.80	–3.09, 0.05	0.060
Perfectionism—Standards	PI vs. Control (T0–T1)	–0.27	0.30	–0.85, 0.31	0.369
	PI vs. Control (T0–T2)	–0.18	0.32	–0.80, 0.45	0.585
	BI vs. Control (T0–T1)	–0.93	0.32	–1.56, –0.31	<0.01
	BI vs. Control (T0–T2)	–0.65	0.36	–1.35, 0.04	0.069
Perfectionism—Discrepancy	PI vs. Control (T0–T1)	–0.19	0.40	–0.98, 0.60	0.633
	PI vs. Control (T0–T2)	–0.09	0.43	–0.94, 0.76	0.838
	BI vs. Control (T0–T1)	–0.56	0.43	–1.41, 0.28	0.196
	BI vs. Control (T0–T2)	–0.77	0.48	–1.71, 0.17	0.113

Note. β = regression estimate; SE = standard error; CI = confidence interval; PI = Podcast Intervention; BI = Blended Intervention; Control = passive control group.

**Table A2:** Planned within-group contrasts from the linear mixed models. Estimates (β), standard errors (SE), 95% confidence intervals (CI), and *p*-values for changes over time within each study group.

Outcome	Group	Comparison	β	SE	95% CI	*p*
Self-compassion	PI	T0–T1	0.31	0.11	0.10, 0.52	<0.01
	PI	T0–T2	0.46	0.11	0.25, 0.68	<0.001
	BI	T0–T1	0.56	0.12	0.32, 0.81	<0.001
	BI	T0–T2	0.60	0.13	0.33, 0.87	<0.001
	Control	T0–T1	0.02	0.10	−0.18, 0.22	0.818
	Control	T0–T2	0.19	0.12	−0.04, 0.42	0.096
Perceived Stress	PI	T0–T1	–0.53	0.48	–1.49, 0.44	0.280
	PI	T0–T2	–1.54	0.49	–2.52, –0.56	<0.01
	BI	T0–T1	–1.57	0.55	–2.67, –0.47	<0.01
	BI	T0–T2	–1.81	0.61	–3.02, –0.60	<0.01
	Control	T0–T1	0.24	0.46	–0.68, 1.15	0.606
	Control	T0–T2	–0.29	0.52	–1.32, 0.75	0.583
Perfectionism—Standards	PI	T0–T1	–0.07	0.22	–0.49, 0.36	0.761
	PI	T0–T2	–0.24	0.22	–0.67, 0.20	0.282
	BI	T0–T1	–0.73	0.25	–1.22, –0.24	<0.01
	BI	T0–T2	–0.72	0.27	–1.25, –0.18	<0.01
	Control	T0–T1	0.20	0.20	–0.20, 0.61	0.326
	Control	T0–T2	–0.06	0.23	–0.52, 0.40	0.787
Perfectionism—Discrepancy	PI	T0–T1	–0.28	0.29	–0.86, 0.30	0.346
	PI	T0–T2	–0.14	0.30	–0.73, 0.45	0.647
	BI	T0–T1	–0.65	0.33	–1.31, 0.01	0.054
	BI	T0–T2	–0.82	0.36	–1.54, –0.09	<0.05
	Control	T0–T1	–0.08	0.28	–0.63, 0.47	0.765
	Control	T0–T2	–0.05	0.31	–0.67, 0.57	0.879

Note. β = regression estimate; SE = standard error; CI = confidence interval; PI = Podcast Intervention; BI = Blended Intervention; Control = passive control group.
